# Tango with Cows

**DOI:** 10.3201/eid1503.000000

**Published:** 2009-03

**Authors:** Polyxeni Potter

**Affiliations:** Centers for Disease Control and Prevention, Atlanta, Georgia, USA

**Keywords:** Art-science connection, emerging infectious diseases, art and medicine, travel and emergence, Liubov Popova, constructivism, cubist futurism, Russian avant-garde, travel-related illness, about the cover

**Figure:**
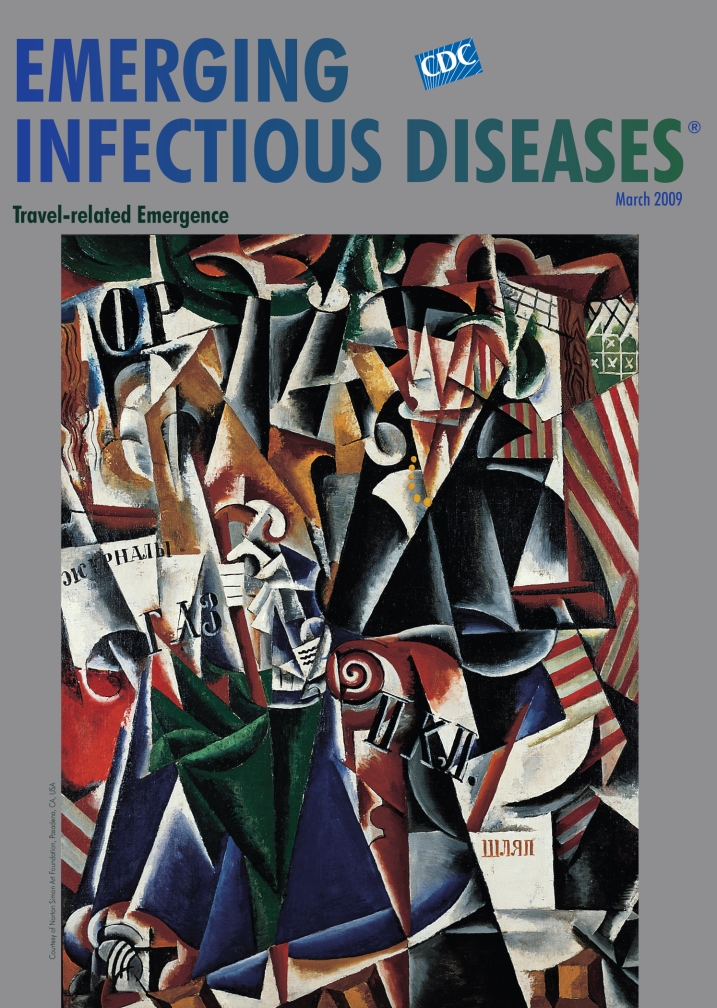
**Liubov Popova (1889–1924) The Traveler (1915).** Oil on canvas (142.2 cm × 105.4 cm) Courtesy of Norton Simon Art Foundation, Pasadena, CA, USA

Farm animals engaged in a sophisticated dance is how poet Vasily Kamensky represented the incongruous entanglement between Russia’s rural past and sweeping modernism. In his daring book Tango with Cows, he abandoned syntax for a spatial arrangement of words on old wallpaper to explore visual poetry. Political oppression, industrial development, and rapid urbanization between the revolutions of 1905 and 1917 shook the foundation of society and promoted experimentation in literature, music, and art. Part of sprouting radical movements known as Russian avant-garde, Liubov Popova made her mark as a leading artist of the 20th century.

Popova was born near Moscow into an affluent family approving of her talent. She traveled widely, within Russia for the architecture and hagiography and abroad to Italy and France. She studied Cubism at the Académie de la Palette under Henri Le Fauconnier and Jean Metzinger. While she admired Giotto and other masters of the Renaissance, her work moved steadily away from naturalism toward a personal style drawn from the flat linearity of Russian icons, the principles of Cubism, and revolutionary ideas. “Representation of reality―without artistic deformation and transformation―cannot be the subject of painting,” she wrote.

In its origins with Picasso and Braque, Cubism was a formal style applied to traditional subjects to depict space and volume through multiple viewpoints and shifting planes. With time, others saw in its geometric precision the potential to capture modern life and its increasing reliance on machines. In Italy, a group called the Futurists used it to express in art what Albert Einstein defined in 1905 in his theory of relativity, a new sense of time, space, and energy. “We wish to exalt aggressive movement,” read the Futurist manifesto, “feverish insomnia, running, the perilous leap, the cuff, and the blow.” From her travels, Popova brought home these influences, which she integrated with folk and decorative elements in shaping the development of combined Cubism and Futurism in Russia.

She joined major art studios and worked with Vladimir Tatlin, advocate of constructivism: the exploration of geometric form in two and three dimensions, not for art’s sake but as service to society. She became increasingly devoted to abstraction, and in 1916 she joined the Supremus group, organized by Kazimir Malevich: “The artist has rid himself of everything which pre-decided the objective ideal structure of life and ‘art,’” he wrote, “He has freed himself from ideas, concepts and representations in order to listen only to pure sensibility.”

Popova turned exclusively to dynamic geometric forms and experimented with texture, rhythm, density, and color in works she called “painterly architectonics.” Unlike the painters of European Cubism and Futurism, who never abandoned recognizable form, she was able to develop a completely nonrepresentational idiom through layered panels of color.

The “Artist Builder,” as she became known, proposed that “Form transformed is abstract and finds itself totally subject to architectonic requirements, as well as to the intentions of the artist, who attains complete freedom in total abstraction, in the distribution and construction of lines, surfaces, volumetric elements and chromatic values.” Popova participated in many exhibitions and became very successful.

In 1921, she joined other artists in rejecting studio painting in favor of industrial design: textile, book, porcelain, ceramic, theater set. As a designer of women’s fabrics at the First State Cotton-Printing factory, she was called upon to “unite the demands of economics, the laws of exterior design, and the mysterious taste of the peasant woman from Tula,” a task she reportedly did not resent, “Not one of her artistic successes ever gave her such deep satisfaction as the sight of a peasant woman and a worker buying lengths of her material.”

Popova’s precipitous rise to artistic prominence was marred by infectious disease catastrophes. Her husband of 1 year, an art historian, died of typhoid fever. Infected herself, she survived but only briefly. She died at age 35 of scarlet fever caught from her son, who died days before she did. Her untimely demise cut short a brilliant artistic career. One obituary read, “This spring, the women of Moscow … the cooks, the service workers―began dressing themselves up. Instead of the former petite bourgeois little flowers, there appeared on the fabrics new and unexpected strong and clear patterns.”

The Traveler, on this month’s cover, was painted when Popova was committed to abstraction but still maintained in her work recognizable forms. At first glance, the image appears a jumble of planes, triangles, cylinders, and semicircles arranged aggressively across the canvas to the very edge. But a closer look yields clues to an image possibly shattered and reconstructed from its fragments.

At the center of the composition, a yellow necklace draws the eye to a hidden female form. Nearby, a collar follows the curve of a cape against a cochleated armrest. The neck, head, and part of a hat are discernible. A green umbrella, firmly clutched, takes front center, its generous flaps against the passenger’s legs and feet below. The seated figure delineated, the viewer can make out passing scenery: a glimpse of railing, a flag, some green. Letters are stenciled over the image forming shop signs and guideposts: “dangerous zone,” “…magazines,” “natural gas.”

Movement is achieved by overlapped planes denoting rapid succession. Shapes, tilted and angled into each other, are shaded and textured for depth and motion. Traveler and surroundings are one, gliding seamlessly in time and space.

Part and parcel of her tumultuous times, Popova recaptured in this painting not just the fragments of a broken image but also revolutionary concepts vital to science and public health. Her traveler, so directly connected with everything, carries with her everything, wherever she goes. And as she moves elegantly from place to place, she changes as does the landscape. She is faster or slower. She picks up things from one place and deposits them in another, ambivalent about past, present, or future.

Just as the rapid influx of technology produced radical art movements, an explosion of travel around the world has irrevocably globalized everything, dragging the rural cow into the metropolitan area to tango. The close meeting of different worlds, back and forth, from country to country and countryside to city, is making the old from the old environment ripe for emergence of the new in a new environment.
